# Chronic thromboembolic pulmonary hypertension: towards an etiology-oriented conceptual framework

**DOI:** 10.3389/fmed.2026.1910552

**Published:** 2026-08-19

**Authors:** Masayuki Nishiyama, Takayuki Okada, Wakana Teranaka, Takahiko Naruko, Tadaaki Koyama

**Affiliations:** 1 Department of Cardiovascular Surgery, Kansai Medical University, Osaka, Japan; 2 Department of Cardiology, Kansai Medical University Medical Center, Osaka, Japan

**Keywords:** balloon pulmonary angioplasty, chronic thromboembolic pulmonary hypertension, classification, pulmonary endarterectomy, pulmonary hypertension

## Abstract

**Background:**

Chronic thromboembolic pulmonary hypertension (CTEPH) is traditionally regarded as a late complication of unresolved acute pulmonary embolism. However, this paradigm is challenged by several observations: up to half of patients lack a documented embolic history, distal or microvascular disease is common, and marked geographic differences in treatment strategies exist. While pulmonary endarterectomy (PEA) predominates in Western centers, Japanese programs have advanced balloon pulmonary angioplasty (BPA), particularly for distal disease. These discrepancies suggest that CTEPH represents a biologically heterogeneous condition rather than a post-embolic entity.

**Methods and conceptual framework:**

We developed an etiology-oriented framework integrating pathogenic mechanisms, vascular lesion distribution, and therapeutic strategies. Using Virchow’s triad as a structural lens, dominant disease drivers were mapped to distinct phenotypes within a thrombo-vasculopathic spectrum.

**Results:**

Five mechanistic phenotypes are proposed: (1) thromboembolism-related (Type Ia), characterized by unresolved embolic obstruction and proximal disease; (2) thrombophilia-associated (Type Ib), driven by systemic hypercoagulability; (3) inflammation- or immune-mediated (Type Ic), associated with endothelial injury and vascular remodeling; (4) structural pulmonary arteriopathy–associated (Type II), in which intrinsic arterial abnormalities precede thrombosis; and (5) idiopathic (Type III), without identifiable cause. These phenotypes form a continuum defined by the relative contributions of thrombotic obstruction and intrinsic vascular remodeling. Within this framework, PEA aligns with proximal disease, whereas BPA and medical therapy correspond to distal vasculopathic phenotypes.

**Conclusion:**

CTEPH should be reconsidered as a heterogeneous thrombo-vasculopathic syndrome. An etiology-based framework linking pathobiology, anatomy, and treatment may help explain global practice variation and support phenotype-guided management.

## Introduction

Chronic thromboembolic pulmonary hypertension (CTEPH) is classified as Group 4 pulmonary hypertension (1). Traditionally, it has been conceptualised as a sequela of unresolved acute pulmonary embolism (PE), resulting in persistent organized thrombotic obstruction of the pulmonary arteries. This paradigm adequately explains patients with proximal, surgically accessible disease. However, several observations suggest that CTEPH may not represent a uniform post-embolic entity. First, epidemiological studies consistently report that 25–50% of patients lack a documented history of acute PE (2). Second, distal and microvascular phenotypes are frequently observed, sometimes disproportionate to the apparent thromboembolic burden (3). Third, marked regional variation in therapeutic strategies has emerged (4): high-volume Western centres predominantly perform pulmonary endarterectomy (PEA), whereas Japanese centres have refined balloon pulmonary angioplasty (BPA), particularly for distal disease (5, 6). These discrepancies may reflect underlying biological heterogeneity rather than differences in clinical philosophy. We therefore propose a conceptual, etiology-oriented framework that seeks to:

1 Clarify mechanistic heterogeneity in CTEPH,
2 Provide a pathobiological context for global therapeutic variation,
3 Stimulate research toward phenotype-guided management.

## Conceptual framework: CTEPH as a thrombo-vasculopathic spectrum

The histological hallmark of CTEPH—organized fibrotic thrombus adherent to the pulmonary arterial wall—should not necessarily be interpreted as evidence of a single initiating mechanism (7). Rather, it may represent a common morphological endpoint arising from multiple upstream processes. Within the classical embolic paradigm, disease progression follows a largely linear sequence: acute embolism → incomplete thrombus resolution → fibrotic organization → mechanical obstruction → secondary distal microvasculopathy (8). While well supported in proximal disease, this sequence does not fully account for patients without embolic history, predominantly distal involvement, or prominent inflammatory vascular remodeling (3). Reframing CTEPH as a thrombo-vasculopathic continuum allows integration of multiple interacting drivers of thrombosis and vascular remodeling, rather than relying on a predominantly embolic model of disease pathogenesis.

## Virchow’s triad as a structural lens

Traditional models emphasise abnormal flow secondary to embolism. The present conceptual approach redistributes dominant pathogenic drivers across the triad, potentially explaining interindividual and geographic variability (9). In this framework, each etiological subtype of chronic thromboembolic pulmonary hypertension can be conceptually mapped to the dominant component of Virchow’s triad. Abnormal blood flow corresponds to embolism-related CTEPH (Type Ia), hypercoagulability to thrombophilia-associated CTEPH (Type Ib), and endothelial injury or chronic inflammation to immune- or inflammation-related CTEPH (Type Ic). In addition, intrinsic structural disturbances of pulmonary arterial flow are represented by structural pulmonary arteriopathy–associated CTEPH (Type II), whereas cases without identifiable etiological drivers are categorized as idiopathic CTEPH (Type III). This conceptual mapping provides a structural framework linking classical thrombogenic mechanisms with the proposed etiological classification.

## Etiology-based classification

The proposed etiology-based classification is summarized in Table 1.

**Table 1:** Etiology-based classification of CTEPH and its pathophysiological and clinical characteristics.

Class classification	Type I CTEPH develops as a consequence of thrombus formation.			Type II: Structural pulmonary arteriopathy	Type III: Idiopathic
	Ia: Thromboembolism	Ib: Thrombophilia	Ic: Inflammation or Immune		
Central pathophysiology	Residual and organized thrombi following acute thromboembolic events	Hypercoagulability with recurrent or sustained thrombus formation	Chronic inflammation, endothelial dysfunction, and impaired fibrinolysis	Structural pulmonary arterial narrowing independent of primary thromboembolism	No identifiable underlying etiology despite comprehensive evaluation
Pathophysiology	Central lesion	Central/peripheral lesion	Central/peripheral lesion	Peripheral lesion	Peripheral lesion
Major predisposing factors	History of PE or DVT Prolonged immobilization, perioperative status, long-term bed rest, reduced mobility due to psychiatric disorders Hormonal therapy (oral contraceptives, hormone replacement therapy) Pregnancy and postpartum period Cancer-associated venous thromboembolism	Antiphospholipid syndrome Inherited thrombophilic disorders (protein C deficiency, protein S deficiency, antithrombin deficiency) Hyperhomocysteinemia Cancer-related hypercoagulable states	Chronic inflammatory diseases (including connective tissue diseases) Chronic infections Malignancy-related chronic inflammation	Peripheral pulmonary artery stenosis Microvasculopathy (small vessel disease) Congenital or acquired intrinsic pulmonary arterial obstruction	Unrecognized or subclinical embolic events Genetic predisposition to impaired thrombus resolution Familial CTEPH
Treatment	PEA highly effective Pulmonary vasodilators	Treatment of the underlying condition. PEA/BPA Pulmonary vasodilators	Treatment of the underlying condition. PEA/BPA (Pulmonary vasodilators)	Pulmonary vasodilators BPA highly effective	Pulmonary vasodilators PEA/BPA

CTEPH, chronic thromboembolic pulmonary hypertension; PE, pulmonary embolism; DVT, deep venous thrombosis; PEA, pulmonary endarterectomy; BPA, balloon pulmonary angioplasty.

### Type Ia: thromboembolism-related CTEPH

#### Dominant pathogenic driver: embolic obstruction and abnormal pulmonary arterial flow

Type Ia represents the classical form of CTEPH. Unresolved thromboemboli from venous thromboembolism (VTE) persist within proximal pulmonary arteries and undergo fibrotic organization, resulting in fixed mechanical obstruction and increased pulmonary vascular resistance (10).

#### Pathobiology

The typical sequence consists of acute pulmonary embolism, incomplete thrombus resolution, collagenized organization, and chronic obstruction (11).

#### Imaging characteristics

Proximal or segmental webs, bands, pouch defects, and occlusions on CTPA or angiography. V/Q scan shows segmental mismatched perfusion defects (12).

#### Common predisposing conditions include

- Prolonged immobilization or long-term bed rest
- Perioperative states
- Reduced mobility related to psychiatric or neurological disorders (13)
- Hormonal therapy (oral contraceptives or hormone replacement therapy) (14)
- Pregnancy and the postpartum period (15)
- Cancer-associated venous thromboembolism (16)

#### Pathological features

Histologically, Type Ia is characterized by organized, collagenized thrombus firmly adherent to and incorporated into the pulmonary arterial wall (10). Recanalization channels are frequently observed. Distal small-vessel arteriopathy may coexist, particularly in advanced cases, reflecting secondary remodeling rather than primary vasculopathy.

#### Therapeutic implications

Type Ia typically manifests as central or surgically accessible disease and is therefore highly amenable to PEA (17). Surgical removal of organized thrombotic material directly addresses the mechanical component of vascular obstruction and remains the treatment of choice when lesions are technically operable (18). However, clinical outcomes after PEA are also influenced by the burden of distal arteriopathy. Patients with substantial distal vascular remodeling may demonstrate persistent pulmonary hypertension despite technically successful removal of proximal obstructive lesions. Accordingly, PEA has demonstrated excellent outcomes in appropriately selected patients, particularly in Western high-volume surgical centers (19). BPA may be considered in patients deemed inoperable or with residual distal disease (20). Lifelong anticoagulation is standard to prevent recurrent thromboembolic events.

### Type Ib: thrombophilia-associated CTEPH

#### Dominant pathogenic driver: hypercoagulability

Type Ib is characterized by systemic prothrombotic states leading to recurrent or persistent thrombosis within the pulmonary vasculature (21).

#### Pathobiology

Unlike the classical embolic paradigm characterized by a single major embolic event, Type Ib is often driven by recurrent or sustained *in situ* thrombosis superimposed on a prothrombotic systemic milieu. Abnormalities in coagulation pathways promote excessive fibrin formation and resistance to endogenous thrombolysis, facilitating thrombus persistence and organization. Representative conditions include elevated factor VIII levels, dysfibrinogenemia, antiphospholipid antibodies, and other inherited or acquired hypercoagulable states (22). Patients may develop CTEPH despite minimal or even subclinical episodes of VTE, suggesting that overt embolism is not always required for disease progression. Thrombi may originate not only from the deep veins of the lower extremities but also from upper extremity veins (e.g., catheter-associated thrombosis) or atypical venous beds in thrombophilic states.

#### Genetic and hemostatic features

An increased prevalence of congenital thrombophilia markers—such as factor V Leiden mutation or prothrombin gene variants—has been reported in certain cohorts, although findings are inconsistent and not universally reproducible. Acquired thrombophilic disorders, particularly antiphospholipid syndrome, appear more consistently associated with CTEPH. For example, Bonderman and colleagues reported antiphospholipid antibodies in approximately 10% of patients with CTEPH, identifying this abnormality as a potential risk factor for disease development (22, 23).

#### Pathological features

Histologically, pulmonary arteries may demonstrate extensive fibrothrombotic material with firm incorporation into the vessel wall. Compared with purely embolic disease, there may be evidence of exaggerated fibrin deposition and impaired fibrinolysis (8). Recurrent thrombosis and organization can lead to layered or multifocal obstructive lesions. As in other subtypes, distal microvascular remodeling may coexist.

#### Patients frequently exhibit identifiable thrombophilic conditions, including

- Antiphospholipid syndrome (24)
- Protein C deficiency (23)
- Protein S deficiency (23)
- Antithrombin deficiency (23)
- Hyperhomocysteinemia (25)
- Cancer-related hypercoagulable states (even in the absence of a clearly documented acute embolic event) (16)

In some cases, no major acute pulmonary embolism is recalled, supporting the concept of cumulative or occult thrombotic injury.

#### Therapeutic implications

Management of Type Ib requires treatment of vascular obstruction and strict control of the underlying hypercoagulable state. Lifelong anticoagulation is essential, tailored to the specific thrombophilia. Anatomical involvement is often mixed; some patients are candidates for PEA, while others require BPA or medical therapy for distal disease. Type Ib represents an intermediate phenotype, driven primarily by systemic hypercoagulability rather than a single embolic event, highlighting the importance of etiologic stratification.

### Type Ic: inflammation- or immune-related CTEPH

#### Dominant mechanism: endothelial injury and chronic inflammation

Although CTEPH has traditionally been regarded as a sequela of unresolved pulmonary embolism, accumulating evidence suggests that a subset of patients may develop the disease through immune-mediated vascular mechanisms. In Japan, disease subtypes associated with specific human leukocyte antigen (HLA) alleles, including HLA-B*5201 and HLA-DPB1*0202, have been reported (26). Associations between HLA polymorphisms and immune-related disorders—particularly connective tissue diseases—are well established. Connective tissue diseases are associated with several forms of pulmonary hypertension, including pulmonary arterial hypertension, interstitial lung disease–related pulmonary hypertension, and chronic thromboembolic pulmonary hypertension (27). These observations support the concept that immune dysregulation and chronic endothelial injury may contribute to CTEPH pathogenesis in selected patients.

#### Pathobiology

In this proposed phenotype, inflammatory and immune mechanisms play a central role in impaired thrombus resolution and aberrant vascular remodeling (23). Persistent endothelial activation, dysregulated inflammatory signaling, and immune cell infiltration have been demonstrated in CTEPH lesions. Histopathologic studies describe intraluminal fibro-inflammatory tissue within organized thrombi, suggesting that vascular obstruction is not merely mechanical but also biologically active. Inflammation may extend beyond the original thrombotic site, promoting progressive remodeling of adjacent and distal vessels.

#### Thrombotic distribution and imaging characteristics

Immune-mediated thrombosis may present as diffuse microthrombi involving small pulmonary arteries and arterioles (28). Consequently, imaging findings often reflect a mixed pattern: proximal organized lesions combined with distal vascular pruning on high-resolution computed tomography or angiography. Perfusion defects may appear heterogeneous and more peripheral, resembling microvascular disease rather than classic central thromboembolic obstruction (29).

#### Genetic and immunologic considerations

Genetic susceptibility may involve polymorphisms affecting inflammatory pathways or endothelial response genes. While HLA-associated predisposition has been reported in certain populations, no single genetic marker has been definitively validated (26). The contribution of immune activation likely represents a multifactorial interaction between host susceptibility and environmental or inflammatory triggers.

This phenotype may be observed in patients with:

- Connective tissue diseases (27)
- Chronic inflammatory disorders (28)
- Chronic infections (30)
- Malignancy-associated chronic inflammation, particularly when endothelial dysfunction predominates (16)

These associations further support the role of sustained inflammatory activation in disease progression.

#### Pathophysiological implications

Endothelial dysfunction, impaired fibrinolysis, and chronic inflammation synergistically promote thrombus organization and progressive vascular remodeling. Although lesions may macroscopically resemble organized thrombi, their biological behavior is strongly influenced by inflammation-driven vascular changes. Microvascular disease is common and may substantially contribute to elevated pulmonary vascular resistance (29).

#### Therapeutic implications

Patients with this phenotype frequently exhibit distal or segmental disease distribution. BPA is often beneficial, particularly in distal lesions. Medical therapy, including pulmonary vasodilators, may play a larger role than in classic surgically accessible CTEPH. However, the potential benefit of pulmonary vasodilators in this phenotype underscores the importance of accurate phenotypic characterization, as inappropriate treatment selection may result in limited efficacy or potentially unnecessary therapy. Therefore, careful assessment of the underlying disease mechanism and lesion distribution is essential before considering phenotype-directed treatment strategies. In contrast, PEA may be less consistently curative when diffuse inflammatory remodeling and microvascular involvement predominate. Notably, this phenotype may be more prevalent in Asian populations, although further multinational studies are required to clarify ethnic and geographic differences.

### Type II: structural pulmonary arteriopathy–associated CTEPH

#### Dominant mechanism: primary structural pulmonary artery disease with secondary thrombosis

While CTEPH is traditionally conceptualized as a consequence of unresolved embolic obstruction, an alternative phenotype may arise from intrinsic structural abnormalities of the pulmonary arteries. In this proposed subtype, fixed luminal narrowing is not solely attributable to organized thrombus but instead reflects primary arterial stenosis, intimal hyperplasia, or abnormal vascular remodeling. Thrombosis, when present, is considered secondary to pre-existing structural narrowing rather than the initiating event.

#### Pathological and mechanistic considerations

Altered vascular remodeling appears central to this subtype. The concept of microvasculopathy (small-vessel disease) has emerged based on several observations:

1 The presence of distal arteriolar lesions resembling those seen in pulmonary arterial hypertension (PAH) (31).
2 Vascular changes in non-thrombotic regions exposed to increased compensatory blood flow.
3 Remodeling distal to obstructive lesions accompanied by bronchial–pulmonary arterial anastomoses (32).

These findings suggest that vascular pathology extends beyond mechanical obstruction and includes intrinsic abnormalities of the pulmonary arterial wall. In Japan, the concept of Peripheral Pulmonary Artery Stenosis (PPS) has been proposed to describe distal-type pulmonary artery stenosis (33). PPS shares certain overlapping characteristics with CTEPH, particularly in therapeutic strategy, further supporting the notion that intrinsic arterial narrowing may constitute a distinct disease phenotype within the broader spectrum of chronic thrombotic pulmonary vascular disorders.

#### Clinical characteristics

This phenotype may include patients with:

- Peripheral pulmonary artery stenosis (33)
- Microvasculopathy (29)
- Congenital or acquired intrinsic pulmonary arterial narrowing
- Non-thromboembolic mechanisms with secondary thrombosis

The initiating event is presumed to be primary structural narrowing rather than embolic obstruction.

#### Pathophysiology

Primary luminal narrowing alters local hemodynamic conditions, including increased shear stress, disturbed flow patterns, and abnormal wall stress, thereby promoting endothelial dysfunction and *in situ* thrombosis with subsequent organization. These hemodynamic alterations may also stimulate distal vascular remodeling and the development of bronchial–pulmonary arterial collateral anastomoses, contributing to progressive pulmonary vascular disease. Repeated thrombotic deposition may mimic classic CTEPH, but unlike embolic disease, the vascular abnormality precedes thrombosis. This entity represents pulmonary hypertension with secondary organized thrombotic lesions due to intrinsic arterial stenosis and is distinguishable from idiopathic PAH by angiographic findings such as webs, bands, and focal stenoses.

#### Therapeutic implications

Distal and segmental involvement is common, often rendering patients suboptimal candidates for PEA. Surgical outcomes may therefore be less predictable compared with classic proximal CTEPH. In contrast, BPA appears particularly well suited to this phenotype, as fixed distal stenoses respond favorably to catheter-based dilation. The high procedural success of BPA reported in Japan may partly reflect a higher prevalence of this intrinsic stenosis–dominant phenotype in Asian cohorts (6). Treatment should also address any identifiable underlying condition contributing to structural arterial narrowing.

### Type III: idiopathic CTEPH

#### Dominant mechanism: undefined

Despite the traditional paradigm that CTEPH represents the sequela of unresolved acute PE, a substantial proportion of patients lack a documented history of VTE, thrombophilia, or overt inflammatory or structural vascular disease. In these individuals, the underlying mechanism of persistent pulmonary hypertension remains unclear. Epidemiological studies suggest that approximately 25–50% of patients diagnosed with CTEPH have no confirmed prior episode of acute PE (2). This observation challenges the exclusively embolic model of disease pathogenesis.

#### Pathobiology

This proposed phenotype encompasses patients in whom no clear etiologic factor—such as acute VTE, inherited thrombophilia, chronic inflammatory disease, or intrinsic pulmonary arterial stenosis—can be identified despite thorough evaluation. Notably, even in the absence of identifiable thrombophilic abnormalities, several studies have suggested a potential genetic predisposition, including familial clustering of CTEPH, supporting the involvement of heritable factors beyond conventional thrombophilia (34, 35).

#### Genetic and pathological considerations

Marked microvascular remodeling resembling pulmonary arterial hypertension in some cases (29).

#### Clinical characteristics

Patients typically exhibit:

- No documented history of thromboembolism
- No identifiable thrombophilia
- No overt inflammatory or structural pulmonary arterial disorder
- Negative results on comprehensive etiologic evaluation

The clinical presentation may otherwise be indistinguishable from other CTEPH phenotypes.

#### Pathophysiological hypotheses

The underlying mechanisms remain speculative. Proposed contributors include:

Unrecognized or subclinical embolic events

- Genetic predisposition to impaired thrombus resolution (36)
- Familial CTEPH (34, 35)
- Abnormal fibrinolytic or endothelial repair pathways
- Primary microvascular remodeling

It is possible that this phenotype represents a heterogeneous group encompassing multiple currently undefined mechanisms.

#### Therapeutic implications

Management is often challenging in cases with an unclear thrombotic etiology. Treatment strategies should therefore be guided primarily by imaging findings, with therapy tailored to the anatomical distribution of lesions. When proximal, surgically accessible disease is present, PEA may be considered, whereas distal or microvascular involvement favors BPA and/or targeted pulmonary vasodilator therapy. This imaging-based approach is essential for optimizing management in this heterogeneous population.

## Discussion

**Figure 1:**
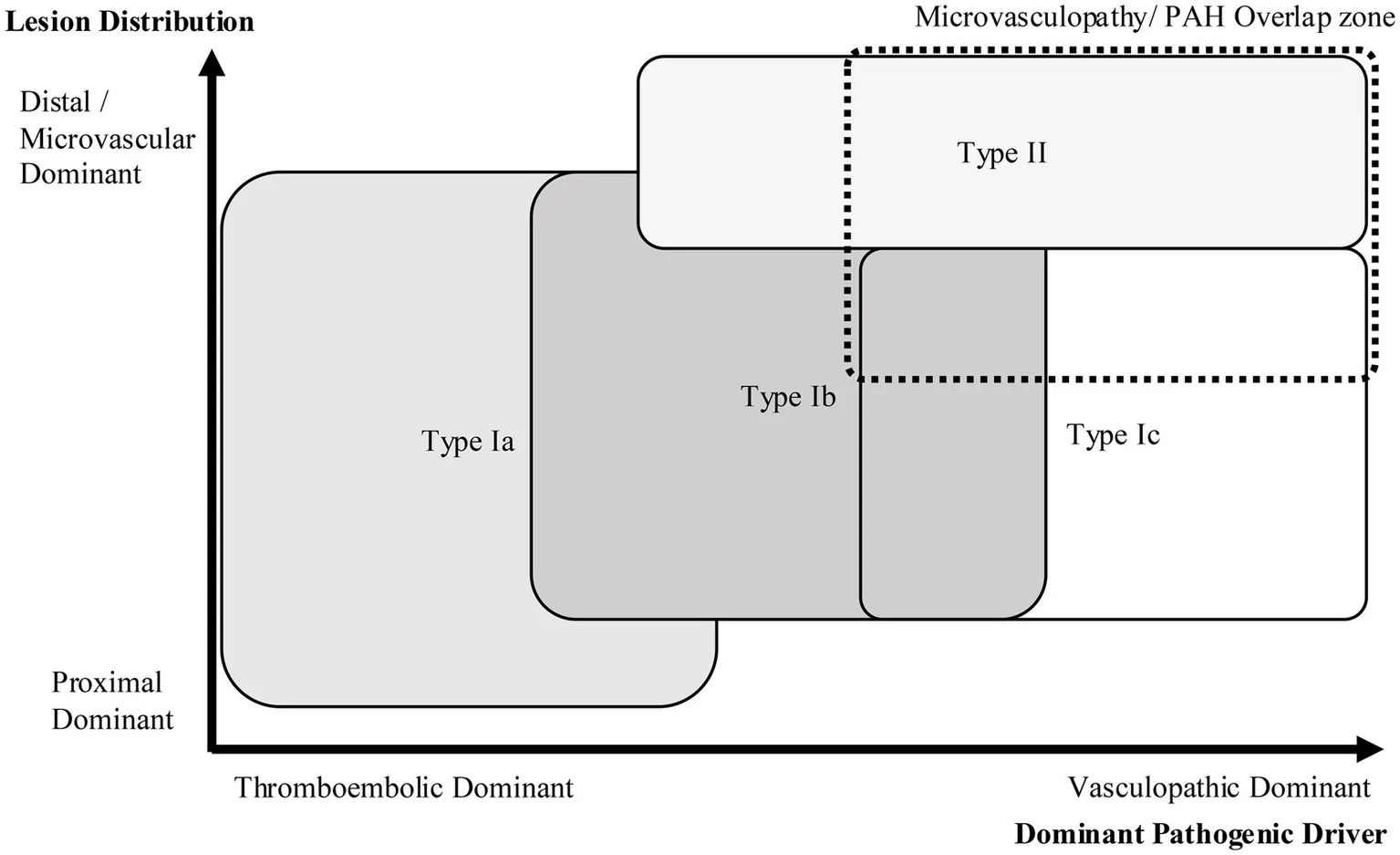
Conceptual distribution of CTEPH phenotypes based on dominant pathogenic driver and anatomical lesion distribution. This conceptual framework illustrates the heterogeneity of CTEPH using two axes: the dominant pathogenic mechanism (horizontal axis) and the anatomical distribution of pulmonary vascular lesions (vertical axis). The horizontal axis ranges from thromboembolic-dominant disease to vasculopathic-dominant mechanisms characterized by intrinsic pulmonary vascular remodeling, while the vertical axis represents the spectrum from proximal pulmonary artery involvement to distal or microvascular disease. CTEPH, chronic thromboembolic pulmonary hypertension; PAH, pulmonary arterial hypertension.

**Figure 2:**
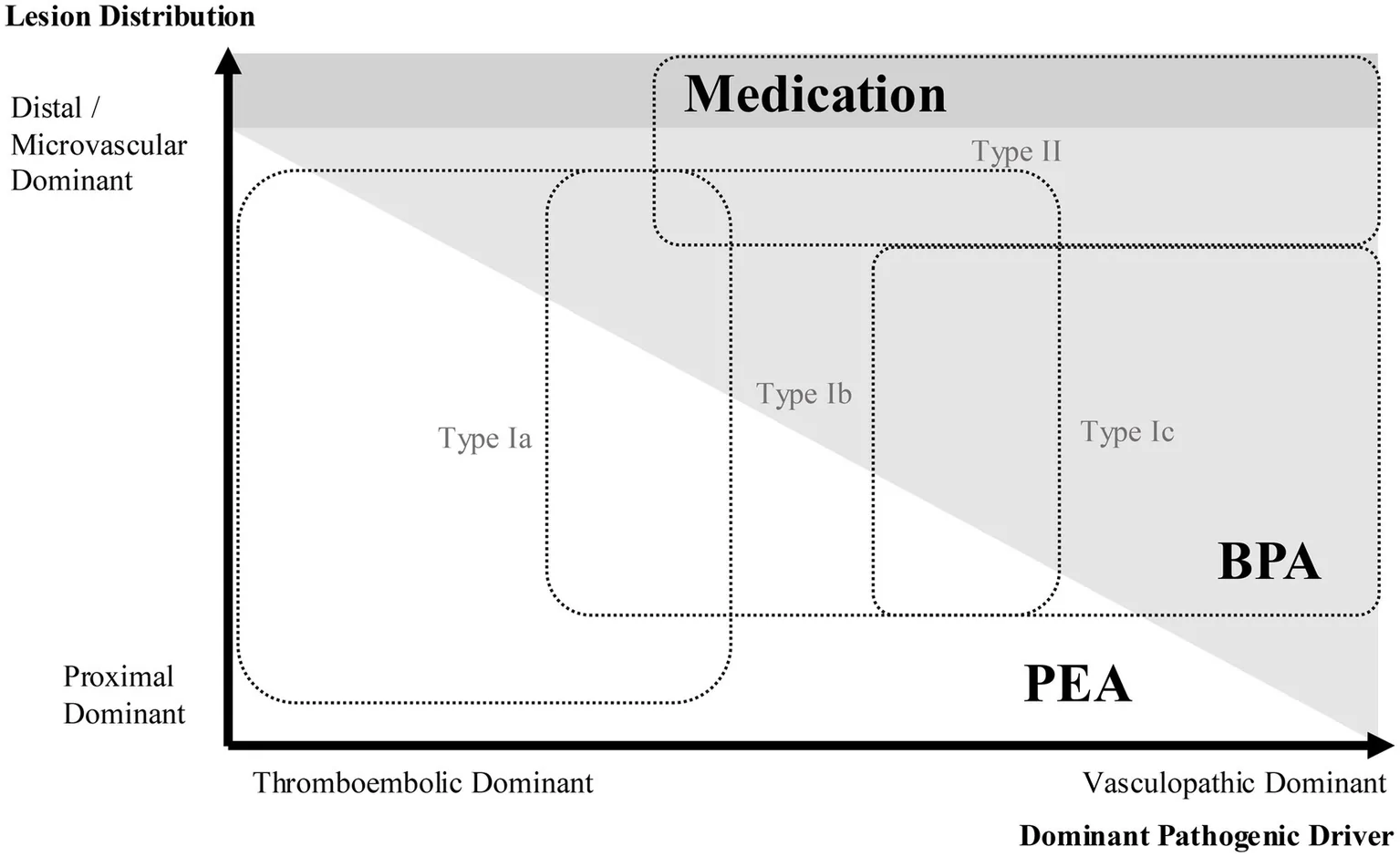
Conceptual therapeutic framework based on lesion distribution and dominant pathogenic mechanism in CTEPH. This schematic illustrates a conceptual treatment strategy for CTEPH based on lesion distribution and dominant pathogenic mechanisms. PEA targets proximal thromboembolic disease, BPA treats distal lesions, and medical therapy addresses microvascular disease or residual pulmonary hypertension, highlighting a complementary multidisciplinary approach. CTEPH, Chronic thromboembolic pulmonary hypertension; PEA, Pulmonary endarterectomy; BPA, Balloon pulmonary angioplasty.

The conceptual frameworks presented in Figures 1, 2 illustrate the heterogeneity of CTEPH by integrating two key dimensions: the dominant pathogenic mechanism and the anatomical distribution of pulmonary vascular lesions. Rather than representing a single embolic disorder, CTEPH can be understood as a spectrum of thrombo-vasculopathic processes in which thrombotic obstruction and pulmonary vascular remodeling contribute to varying degrees (1). In Figure 1, disease subtypes are positioned along two axes. The horizontal axis represents the dominant pathogenic driver, ranging from thromboembolic-dominant mechanisms to vasculopathic-dominant processes characterized by intrinsic pulmonary vascular remodeling. The vertical axis reflects the anatomical distribution of lesions, from proximal pulmonary artery involvement to distal or microvascular disease. Within this framework, Type Ia occupies the region defined by thromboembolic dominance and proximal disease, corresponding to the classical paradigm in which unresolved pulmonary embolism leads to organized fibrotic thrombus within the proximal pulmonary arteries (37). However, clinical observations suggest that this model does not explain all cases of CTEPH (38). A substantial proportion of patients lack a documented history of acute pulmonary embolism, and distal-dominant lesions or inflammatory vascular remodeling are frequently observed. Type Ib represents an intermediate phenotype in which thrombophilic conditions promote recurrent or persistent thrombosis affecting both proximal and distal pulmonary arteries. Type Ic extends further toward the vasculopathic spectrum and includes cases associated with chronic inflammation or immune-mediated endothelial injury, where vascular remodeling may precede or promote thrombosis. Type II represents structural pulmonary arteriopathy involving intrinsic abnormalities of the pulmonary arterial architecture, often affecting distal vessels and overlapping with microvasculopathy observed in pulmonary arterial hypertension (39). This framework can also be interpreted through Virchow’s triad. Whereas traditional concepts emphasize abnormal flow caused by embolic obstruction, the present model distributes the dominant drivers across the triad: flow obstruction (Type Ia), hypercoagulability (Type Ib), endothelial injury and inflammation (Type Ic), and intrinsic structural abnormalities affecting vascular flow (Type II). Such a structure may better explain the clinical variability observed among patients with CTEPH. Figure 2 extends this concept by linking pathophysiological heterogeneity to therapeutic strategies. PEA is positioned in the region characterized by proximal lesions and thromboembolic dominance, where organized thrombus in the central pulmonary arteries represents the primary surgical target (4). In contrast, BPA is located toward the distal and more vasculopathic portion of the framework, reflecting its role in treating segmental or subsegmental pulmonary arterial lesions that are not amenable to surgical removal. In the upper region of the model, representing distal microvascular disease, medical therapy—including lifelong anticoagulation and pulmonary vasodilators—plays an important role, particularly in patients with predominant microvasculopathy or residual pulmonary hypertension after mechanical intervention (40, 41).

Importantly, this framework may also help explain geographic differences in treatment strategies. Western centers have historically reported excellent outcomes with PEA in proximal thrombus-dominant disease, whereas Japanese centers have refined BPA for distal phenotypes (5, 42). These differences may therefore reflect variations in disease phenotype rather than differences in therapeutic philosophy. In summary, the conceptual models presented in Figures 1, 2 highlight CTEPH as a heterogeneous thrombo-vasculopathic syndrome defined by the interaction between thrombosis and pulmonary vascular remodeling. Integrating pathogenic mechanisms, anatomical lesion distribution, and treatment strategies within a unified framework may facilitate a more comprehensive understanding of the disease and support more individualized management approaches. Type III should be distinguished from chronic thromboembolic disease (CTED), or more recently termed chronic thromboembolic pulmonary disease (CTEPD) without pulmonary hypertension. While both entities share persistent thromboembolic vascular lesions, Type III is characterized by established resting pulmonary hypertension despite the absence of identifiable etiological drivers. In contrast, CTED/CTEPD without pulmonary hypertension represents patients with chronic thromboembolic lesions who have not yet developed resting pulmonary hypertension. Thus, these entities differ primarily in hemodynamic status rather than etiology. Nevertheless, progression from CTED/CTEPD to Type III through progressive distal vasculopathy or microvascular remodeling remains biologically plausible, although this hypothesis has not yet been demonstrated and warrants prospective investigation.

### Therapeutic implications across phenotypes

The proposed classification may also have therapeutic implications by aligning treatment strategies with dominant pathogenic mechanisms (Table 1). Type Ia, characterized by proximal organized thromboembolic obstruction, is generally the phenotype most amenable to PEA, although postoperative outcomes remain influenced by the burden of distal arteriopathy. In contrast, Type Ib and Type Ic frequently exhibit mixed proximal and distal involvement, requiring a combination of treatment directed at the underlying disease process, mechanical intervention (PEA or BPA), and in selected cases pulmonary vasodilator therapy. Accurate phenotypic characterization is particularly important in Type Ic, where inflammatory vasculopathy and microvascular disease may increase the relevance of medical therapy but also raise the risk of inappropriate treatment selection. Type II, driven primarily by intrinsic pulmonary arterial stenosis and structural vascular remodeling, may be especially suitable for BPA because of its predominantly distal distribution. Finally, Type III frequently lacks surgically accessible lesions and may require an individualized approach combining BPA and medical therapy. Importantly, therapies that are highly effective in one phenotype may be less beneficial in another, emphasizing the need for a more personalized treatment strategy based on the dominant pathogenic mechanism rather than anatomical lesion distribution alone.

## Conclusion

CTEPH should be viewed not as a uniform post-embolic disease, but as a heterogeneous thrombo-vasculopathic syndrome with multiple etiologically distinct subtypes. Identifying dominant pathogenic drivers may better align biology with therapy, explain regional treatment differences, and support precision medicine approaches. Multicenter studies are needed to validate this framework and assess whether etiology-guided management improves long-term outcomes.

## Limitations

This proposal is hypothesis-generating. Objective criteria for subtype classification are not yet established, and pathological confirmation is largely limited to surgically treated patients, introducing potential selection bias. Distinguishing primary intrinsic stenosis from secondary remodeling remains challenging with current imaging modalities. Category overlap is inevitable, and dominant-driver classification requires consensus definitions. Prospective multicenter validation integrating imaging, molecular data, and longitudinal outcomes is needed before clinical implementation.
